# Functional Starters for Functional Yogurt

**DOI:** 10.3390/foods4010015

**Published:** 2015-02-05

**Authors:** Mattia P. Arena, Graziano Caggianiello, Pasquale Russo, Marzia Albenzio, Salvatore Massa, Daniela Fiocco, Vittorio Capozzi, Giuseppe Spano

**Affiliations:** 1 Department of Agricultural, Food and Environmental Sciences, University of Foggia, Via Napoli 25, Foggia 71122, Italy; E-Mails: mattiapia.arena@unifg.it (M.P.A.); graziano.caggianiello@unifg.it (G.C.); pasquale.russo@unifg.it (P.R.); marzia.albenzio@unifg.it (M.A.); salvatore.massa@unifg.it (S.M.); vittorio.capozzi@gmail.com (V.C.); 2 Promis Biotech s.r.l., Via Napoli 25, Foggia 71122, Italy; 3 Department of Clinical and Experimental Medicine, University of Foggia, Via Pinto 1, Foggia 71122, Italy; E-Mail: daniela.fiocco@unifg.it

**Keywords:** yogurt, riboflavin, multifunctional strains, probiotic

## Abstract

In this study, we investigated the multifunctionality (microbial starters and probiotics) of *Lactobacillus plantarum* WCFS1 and *Lactobacillus plantarum* CECT 8328 strains used as microbial starters for the production of yogurt in combination with *Lactobacillus delbrueckii* ssp. *bulgaricus* and *Streptococcus thermophilus*. The ability of the probiotic strains to survive oro-gastrointestinal stresses was monitored by an *in vitro* assay simulating the human digestive tract. The transcriptional level of several genes involved in the immune response suggested that the probiotic strains may have a favorable influence on immunomodulation. Overall, this study revealed that the tested *Lactobacilli* exhibited suitable technological features for yogurt production and might be used to formulate novel food with immunomodulating effects.

## 1. Introduction

Lactic acid bacteria (LAB) are traditionally used to obtain fermented food from different raw materials, e.g., milk, vegetable, cereal and meat products [1,2]. Moreover, several strains of LAB are able to confer health benefits to humans [3], presenting probiotics features, e.g., reinforcement of the intestinal epithelial barrier, reduction of allergies, antagonism against pathogens and modulation of gut-associated lymphoid tissue (GALT) immune activity [4,5,6,7]. The human gastrointestinal system fulfils the important role of digesting and absorbing the molecules ingested through the diet required for cell growth and activity. Therefore, it is the principal interface between human tissues and external factors, such as food and microorganisms [8]. The human gastrointestinal system acts also as a central component of the immune system, performing both the function of a physical barrier and producing different types of immune mediators, *i.e*., cytokines, which have the role of initiating, sustaining and modulating the immune response against injury, microbial attacks and other antagonistic stimuli [9,10]. However, immune homeostasis, that is the maintaining of a well-balanced ratio between pro- and anti-inflammatory processes [11,12], can be compromised, in particular inflammatory gut diseases, such as Crohn’s disease (CD) and ulcerative colitis (UC), in the case of stress conditions, in allergic diseases and due to aging factors [13,14,15]. Some diet components have been shown to modulate and/or maintain immune homeostasis [16]. Similarly, one of the most important actions on the gut immune system of probiotic bacteria is the ability to influence the genes coding for cytokines, affecting the function of various immune parameters by immuno-stimulation and/or immunomodulation action.

Haller *et al*. [17] demonstrated that a strain of *Lactobacillus sakei* was able to induce the transcriptional level of genes coding for pro-inflammatory cytokines, *i.e*., IL-1β, IL-8 and apoptosis inducer TNF-α. Likewise, *Lactobacillus helveticus* and *Lactobacillus acidophilus* have been shown to have the capability to enhance the production of cytokines IL-6 by intestinal epithelial cells [4,18]. Moreover, Grimoud *et al*. [19] demonstrated that the anti-inflammatory properties of several probiotic bacteria, including strains of *Bifidobacterium bifidum*, *Lactobacillus acidophilus*, *Lactobacillus lactis* and *Lactobacillus plantarum*, were able to reduce IL-8 secretion in stimulated intestinal cells. Interestingly, it was also proven that *Lactobacillus rhamnosus* GG inoculated in milk significantly downregulated the gene expression of CR1, CR3, FcγRIII and FCαR, important phagocytosis receptors that are highly expressed in milk-hypersensitive patients [20]. Ogawa *et al.* [21] showed a symbiotic influence of the probiotic bacteria, *Lactobacillus casei* ssp. *casei*, and dextran to enhance the efficiency of IL-15 production.

Consequently, behind a growing consumer demand for health-promoting and disease-preventing food, the investigation on microorganisms with both technological and probiotic characteristics has increased in recent years, aiming to develop novel products with higher physical, chemical, technological and functional quality.

The aim of this study was to investigate the potential immunomodulatory effects of *Lactobacillus plantarum* WCFS1 and *Lactobacillus plantarum* CECT 8328 strains inoculated into yogurt fermented with *Lactobacillus delbrueckii* ssp. *bulgaricus* and *Streptococcus thermophilus*. Differences in terms of pH, the content of lactic acid, fat, proteins, casein, nitrogen fractions and peptide profiles were evaluated in yogurt during storage at 4 °C. Furthermore, we carried out the microbiological analysis of 7-, 14-, 21- and 28-day stored yogurts in order to monitor the capability of strains to persist in the dairy product during a simulated period of shelf life. Finally, the ability of the *Lactobacillus* strains tested to survive in the human digestive tract and their effects on the transcriptional level of several genes involved in the immune response using LPS-stimulated monocytoid THP-1 cells as a model were analyzed.

## 2. Results and Discussion

### 2.1. Chemical Analysis

The chemical composition of milk used in all experiments was determined prior to the fermentation processes and was as follows: fat 3.6% ± 0.1%, protein 3.3% ± 0.2%, lactose 4.7% ± 0.1% and casein 2.5% ± 0.1%. Furthermore, the yogurt samples were analyzed for their pH, lactic acid, protein, casein, nitrogen fractions, fat content and peptide profile in order to investigate the influence by different strains of *L. plantarum* on yogurt fermentation over 1, 14 and 28 days of storage at 4 °C (Table 1).

**Table 1:** Chemical composition of yogurt.

Stain	pH	Prot (%)	Casein (%)	WSEs (%)	Fat (g/100 g)	Lactic acid (g/L)
*1 Day*						
CNT	4.19 ± 0.01	3.28 ± 0.11	2.45 ± 0.11	0.15 ± 0.01	2.68 ± 0.17	3.98 ± 0.35
LpWCFS1	4.07 * ± 0.02	3.43 ± 0.11	2.41 ± 0.11	0.19 ± 0.01	3.75 * ± 0.39	4.43 ± 0.65
Lp8328	4.28 * ± 0.01	3.35 ± 0.23	2.59 ± 0.16	0.19 ± 0.01	4.05 * ± 0.13	4.65 ± 1.04
*14 Days*						
CNT	4.25 ± 0.01	3.03 ± 0.00	2.42 ± 0.00	0.11 ± 0.01	4.43 ± 0.10	4.90 ± 0.81
LpWCFS1	4.18 * ± 0.01	2.95 ± 0.11	2.34 ± 0.11	0.10 ± 0.00	4.48 ± 0.10	5.05 ± 0.04
Lp8328	4.13 * ± 0.01	3.19 ** ± 0.00	2.27 ± 0.14	0.12 ± 0.00	4.45 ± 0.06	5.43 ± 0.51
*28 Days*						
CNT	4.22 ± 0.01	1.99 ± 0.11	1.61 ± 0.07	0.10 ± 0.00	4.3 ± 0.20	5.14 ± 0.23
LpWCFS1	4.17 * ± 0.01	2.87 * ± 0.00	2.24 * ± 0.02	0.10 ± 0.00	4.4 ± 0.00	5.46 ± 0.62
Lp8328	4.21 ± 0.01	2.95 * ± 0.11	2.13 * ± 0.11	0.12 ± 0.00	4.3 ± 0.20	5.44 ± 0.26

Prot, Protein content; WSE, water-soluble extracts; CNT, positive control fermented with *S. thermophilus* and *L. delbrueckii* ssp. *bulgaricus*; LpWCFS1, yogurt fermented with *S. thermophilus*, *L. delbrueckii* ssp. *bulgaricus* and *L. plantarum* WCFS1; Lp8328, yogurt fermented with *S. thermophilus*, *L. delbrueckii* ssp. *bulgaricus* and *L. plantarum* CECT 8328. Values represent the mean ± the standard deviation (SD). Statistical analyses were carried out by the Student’s *t*-test, and significant differences are relative to the control sample (* *p* < 0.05 and ** *p* < 0.005).

The results showed that the pH values of the control yogurt (fermented only by starter strains without any inoculation with *L. plantarum* strains) were 4.19, 4.25 and 4.22 after 1, 14 and 28 days of storage, respectively. The yogurt samples inoculated with *L. plantarum* WCFS1 and *L. plantarum* CECT 8328 presented pH values after 1 and 14 days of storage significantly different from the control. However, these differences disappeared after 28 days of storage for *L. plantarum* CECT 8328, while for the yogurt inoculated with *L. plantarum* WCFS1, the pH values remained significantly lower (pH 4.17), even after 28 days. Frequently, the pH of yogurt drops during storage, the so-called post-acidification problem, and this can lead to a loss of organoleptic quality. Commonly, consumers prefer yogurts presenting mild acidity (pH 4.2–4.4); thus, microbial cultures with a mild acid production ability are usually selected in order to obtain yogurts with mild acidity and pH stability during shelf-life [22,23]. Interestingly, all of our strains, once fermentation had been carried out during yogurt production, did not cause further lowering of pH in the yogurt samples over the entire storage time.

The protein fraction was also quantified, and as the average, its content was around 3.43% and 2.95% after 1 day and 14 days, with no significant differences among the collected samples. However, a higher percentage of protein (3.19%) was observed, after 14 days, for the yogurt inoculated with *L. plantarum* CECT 8328. Nevertheless, after 28 days of storage, the protein contents of all yogurts inoculated with *L. plantarum* strains were significantly higher (2.87% and 2.95% for *L. plantarum* WCFS1 and *L. plantarum* CECT 8328) than the amount measured in the control sample (1.99%).

The percentage of casein decreased in a time-dependent manner for all of the samples analyzed. However, the ability to degrade casein was reduced in the yogurt inoculated with *L. plantarum* WCFS1 and *L. plantarum* CECT 8328 compared to the control sample.

The total soluble nitrogen content decreased during the storage, possibly due to the proteolytic activity of bacteria, without any significant differences between the trials. Similarly, no significant differences were observed for fat and lactic acid content over medium and long storage times. As the average, the lactic acid content was 4.51, 5.01 and 5.1 g/L after 1, 14 and 28 days, respectively.

Overall, the results reported suggested that the yogurts fermented with *S. thermophilus* and *L. delbrueckii* ssp. *bulgaricus* co-inoculated with *L. plantarum* strains led to the final product showing a different pH value over small and medium, but not long, storage times. Moreover, the inoculated samples presented higher protein and casein content compared to the control. Conversely, the percentage of water-soluble extracts (WSEs) and fat and the lactic acid amount after 14 and 28 days were similar to the control.

**Figure 1:**
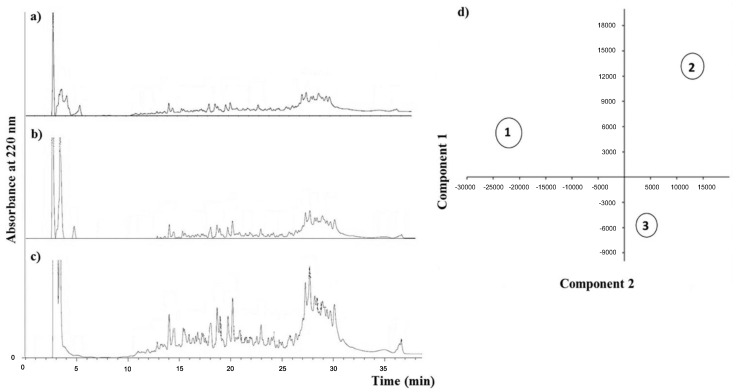
Representatives RP-HPLC profiles of the water-soluble extracts (WSEs) and PCA obtained from the yogurt studied in this work. The chromatograms are relative to yogurt fermented with only *S. thermophilus* and *L. delbrueckii* ssp. *bulgaricus* (CNT) after 1 (**a**), 14 (**b**) and 28 (**c**) days of storage. PCA plots (**d**) were based on the analysis of all peptide profiles of all yogurt samples. CNT, 1; *L. plantarum* WCFS1, 2; *L. plantarum* CECT 8328, 3.

During the milk fermentation, lactic acid bacteria are involved in casein proteolysis in order to provide the amino acids and peptides needed for their growth. Thus, the molecule accumulation in the final fermented product depends on the hydrolase pathways possessed by selected strains of bacteria. Consecutively, the peptide profile may influence the nutrition quality of the fermented product and may condition the growth of other co-inoculated microorganisms [24]. For instance, it is known that the gradual degradation of peptides by the yogurt starter *L. bulgaricus* cultures promotes the growth of *S. thermophilus*, which more rapidly produces lactic acid [25,26]. Here, we analyzed the WSE during the storage of yogurt fermented with different *Lactobacilli* strains co-inoculated with the two yogurt starter cultures, *S. thermophilus* and *L. bulgaricus* subsp. *delbrueckii*. The results from RP-HPLC (Figure 1a–c) showed a basically similar peptide profile for all treatments with quantitative differences for the peptide content of the water-soluble extracts over storage time (increasing in a time-dependent manner). Compared with the control, *L. plantarum* CECT 8328 was the most proteolytic strain. Principal component analysis (PCA) was used to analyze the clustering of the treatments. As reported in Figure 1d, the peptide profiles of *L. plantarum* CECT 8328 and *L. plantarum* WCFS1 were different from those observed for the control samples.

### 2.2. Viability of Lactobacillus plantarum Strains

We used the Q-PCR-propidium monoazide (PMA) methodology to investigate the viability of *L. plantarum* strains. The use of PMA associated with Q-PCR has been shown to be valuable for discriminating between live and dead microorganisms [27]. PMA selectively penetrates the membranes of dead cells and links the dsDNA. The dsDNA-PMA complex can be activated by light and bind the cellular hydrocarbon moiety to form highly-stable compounds. The dsDNA-PMA-hydrocarbon complex is left out during Q-PCR amplification; thus, the DNA of dead cells is not detected.

**Figure 2:**
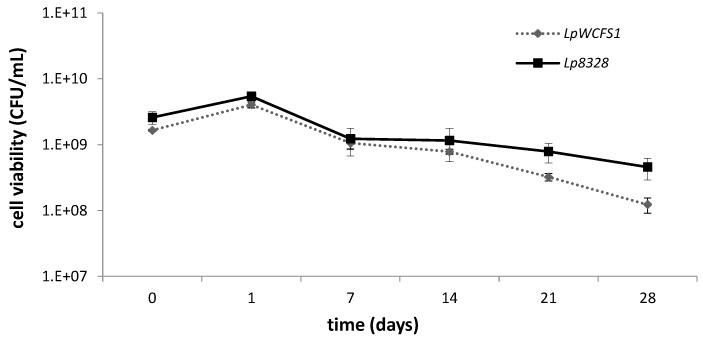
Cell viability of *L. plantarum* strains used to produce yogurt at Time 0 (initial inoculum prior to the start of the fermentation), 1, 14, 21 and 28 days of storage at 4 °C. LpWCFS1, *L. plantarum* WCFS1; Lp8328, *L. plantarum* CECT 8328. Values represent the mean ± the standard deviation (SD) of two different experiments.

In this study, the viability of *L. plantarum* strains for every yogurt sample was analyzed at Time 0 (initial inoculum prior to the start of the fermentation), 1, 14, 21 and 28 days of storage. As shown in Figure 2, the CFU/mL of each *Lactobacilli* strain decreased in a time-dependent manner with no significant differences after 1, 7 and 14 days. However, we observed a significantly higher cell survival for the vitamin over-producing strain, *L. plantarum* CECT 8328, after 21 and 28 days with respect to *L. plantarum* WCFS1. Overall, microbial counts during the storage were higher than 10^8^ CFU/mL, according to the probiotic recommended threshold to confer beneficial effects to humans [28].

### 2.3. Oro-Gastrointestinal Tolerance Assay

The capability of *L. plantarum* WCFS1 and *L. plantarum* CECT 8328 inoculated into yogurt matrix to endure human digestion was also investigated by an *in vitro* simulation of the gastrointestinal tract (oral, gastric and intestinal conditions) [29].

**Figure 3:**
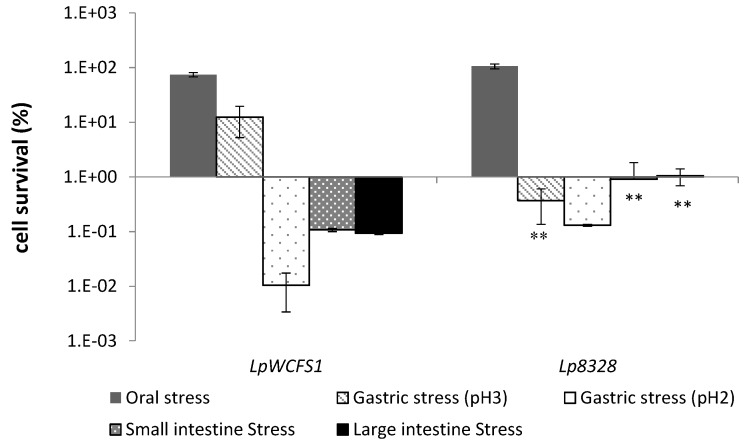
Bacterial survival after exposure of *L. plantarum* strains inoculated in yogurt to an *in vitro* model simulating the human oro-gastrointestinal tract (oral, gastric pH 3.0 and pH 2.0 and small and large intestine stresses). Viability was calculated as the percent of survival relative to the untreated control (*i.e*., the sample before the simulated digestion). Values represent the mean ± the standard deviation of three different experiments. Statistical analyses were carried out by the Student’s *t*-test, and significant differences are relative to the probiotic *L. plantarum* WCFS1 strain (* *p* < 0.05 and ** *p* < 0.005). LpWCFS1, *L. plantarum* WCFS1; Lp8328, *L. plantarum* CECT 8328.

The results indicated variable survival percentages depending on the strains and the gastrointestinal stress steps (Figure 3). The bacterial percentage of survival with respect to untreated samples was not influenced by oral stress. Conversely, the persistence of *Lactobacilli* strains was mainly affected under gastric conditions in a pH-dependent manner, similar to the results obtained by other authors [29,30,31]. These findings are correlated with the greater difficulty of bacteria to resist low pH and underlined the necessity to select probiotic bacteria with a strong ability to tolerate acid environments in order to overcome the gastric sector and reach the intestine. The percentage of cell survival of *L. plantarum* WCFS1 and *L. plantarum* CECT 8328 after exposure to gastric stress at pH 3.0 decreased by about 1 log unit and 2.5 log units, respectively. However, the percentage of survival of *L. plantarum* CECT 8328 was significantly higher than that of *L. plantarum* WCFS1 after gastric stress at pH 2.0 and under intestinal stresses (Figure 3).

### 2.4. Stimulation of THP-1 Cells with Lactobacillus plantarum Strains

**Figure 4:**
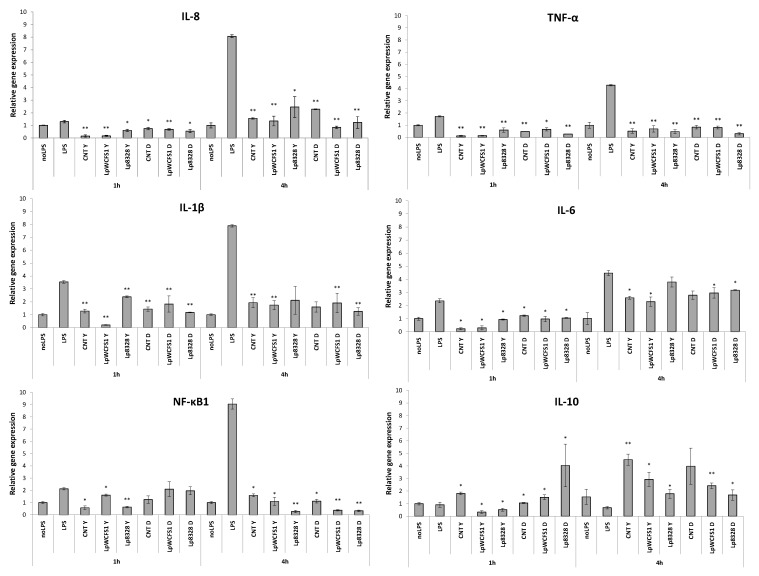

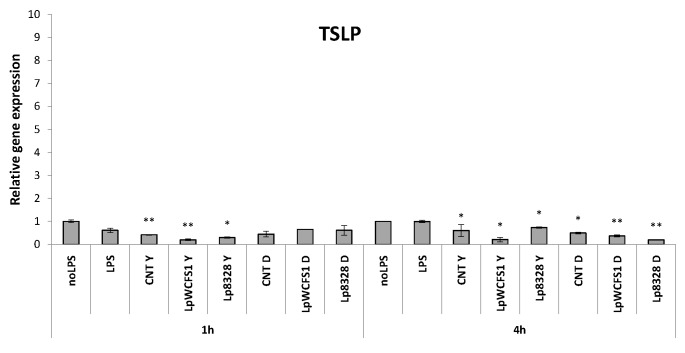
Relative expression of cytokine-related genes after the exposure to undigested (Y) and *in vitro* digested (D) samples containing *Lactobacilli* strains over 1 h and 4 h of treatments. Values represent the mean ± the standard deviation of two different experiments. Statistical analyses were carried out by the Student’s *t*-test, and significant differences are relative to LPS-stimulated THP-1 cells without strains (LPS, positive control) (* *p* < 0.05 and ** *p* < 0.005). noLPS, no LPS-stimulate THP-1 cells used as negative control; CNT, yogurt inoculated with *S. thermophilus* and *L. bulgaricus* subsp. *delbrueckii* was the matrix control; LpWCFS1, yogurt inoculated with *S. thermophilus*, *L. bulgaricus* subsp. *delbrueckii* and *L. plantarum* WCFS1; Lp8328, yogurt inoculated with *S. thermophilus*, *L. bulgaricus* subsp. *delbrueckii* and *L. plantarum* CECT 8328.

The human gastrointestinal system fulfils the important role of metabolizing and absorbing the nutrients indispensable for cell growth and activity. Furthermore, the gut acts also as a central component of the immune system, both performing the function of a physical barrier and producing different types of immune mediators essential to maintaining the immune homeostasis [9,10]. However, malfunctions of immune homeostasis with continuous release of pro-inflammatory cytokines can result in a persistent inflammation process that can lead to intestinal dysfunction, cell damage and chronic inflammatory gut disease, such as Crohn’s disease (CD) and ulcerative colitis (UC), as well as several multiple disease states [12,32]. The capability of diet components, including functional food containing probiotic bacteria, to modulate the immune-related genes could ameliorate and/or prevent the intestinal inflammatory conditions. In this context, the potential ability of probiotic strains inoculated in yogurt to influence the expression level of genes involved in immunomodulation was investigated. Since food consumed by diet is exposed to several digestive steps to be metabolized, we carried out the assays exposing THP-1 cells to both untreated and *in vitro* digested yogurt samples containing *L. plantarum* strains, in order to understand whether the *in vitro* digestion could affect the immunomodulation activity. The components of bacterial cell walls, peptidoglycan (PG), present in both Gram-positive and Gram-negative bacteria, and lipopolysaccharide (LPS), present in Gram-negative microorganisms, may stimulate the human cells in a receptor-dependent process activating the release of several immune mediators. For instance, LPS-activated macrophages can produce cytokines, such as interleukins (IL-8, IL1β, IL-6) and/or tumor necrosis factor-α (TNF-α), involved in the immune response [33]. For this reason, we exposed the differentiated THP-1 cells to only LPS (positive control) and LPS-sample in order to compare the transcriptional level of several genes involved in the regulation of the immune response, *i.e*., IL-8, TNF-α, IL1β, TSLP, IL-6, NF-κB1 and IL-10. Cytokines play a crucial role in the inflammatory process, being able to coordinate the initiation, amplification and interruption of immune response [16]. As shown in Figure 4, the relative expression of genes involved in cytokine-mediated signaling was globally affected by exposure to the sample containing *Lactobacilli* in a strain- and time-dependent manner. The transcriptional levels of IL-8 were significantly reduced by all samples, both undigested and *in vitro* digested samples. We observed a slightly higher ability of undigested samples to moderate the transcriptional level of IL-8 gene after 1 h of incubation with respect to the *in vitro* digested samples. Conversely, this trend was not noted after 4 h of exposure, where the *in vitro* digested samples containing *Lactobacilli* were mostly able to moderate the expression of IL-8. IL-8, a potent cytokine, is involved in host defense by the activation and chemo-attraction of neutrophils. High levels of IL-8 are associated with inflammatory diseases and conditions, such as asthma, inflammatory bowel disease (IBD) and exposure to LPS of Gram-negative bacteria [34]. In this study, the transcriptional analysis showed that the probiotic bacteria inoculated in food matrix were able to downregulate the gene expression of IL-8.

The strongest activators of the IL-8 gene are the pro-inflammatory cytokines, tumor necrosis factor-α (TNF-α) and interleukin 1β (IL-1β) [34]. Moreover, TNF-α is able to coordinate the enhancement of the inflammatory response by activating neutrophils, mononuclear phagocytes and other cell types, such as eosinophils [33,35,36]. IL-1β belongs to the IL1 family, which plays an important role in the cascade response of the innate immune system, incrementing cytokine production in dendritic cells, stimulating phagocytosis in macrophages and increasing oxidative burst and protease release in neutrophils, which promote the differentiation and proliferation of T-cells [37]. In the case of TNF-α gene analysis, our results showed a significant reduction of expression both after 1 h and 4 h of exposure for all treatments with samples containing probiotic bacteria without significant differences between undigested and *in vitro* digested trials. Similarly, we found a downregulation of the transcriptional levels of the IL-1β gene after 1 h and 4 h of incubation.

Another multifunctional cytokine implicated in pro- and in several anti-inflammatory processes is interleukin 6 (IL-6), which is highly produced in response to pathogen infections, the growth of normal and neoplastic cells and also after LPS-induction [4]. In this study, the IL-6 gene expression was significantly reduced by exposure to all samples containing *Lactobacilli* with respect to the control (only LPS). However, we observed that the transcription level of IL-6 was more quickly decreased after a shorter time of exposure (1 h) compared to longer incubation with probiotics (4 h). Moreover, we showed a marginally higher capacity of undigested samples to reduce IL-6 gene expression with respect to the *in vitro* digested samples after 1 h. Contrariwise, this trend was not noted after 4 h of exposure, because the *in vitro* digested samples inoculated with *Lactobacilli* were mostly able to moderate the expression of IL-6, similarly to the results obtained for IL-8 gene analysis. These results are in agreement with previous studies by Riedel *et al.* [38], where strains of *Bifidobacterium longum* and *Bifidobacterium bifidum* were shown to reduce the transcriptional level of IL-8 and TNF-α genes in the HT-29 cell line. Other authors demonstrated that several probiotic microorganisms induced a dramatic reduction of the secretion of IL-8 protein in HT-29 cells, highlighting their anti-inflammatory effects [19,39]. Furthermore, strains belonging to *Kluyveromyces*, *Lactobacillus* and *Bifidobacterium* genera were shown to determine the decrease in the secretion level of the pro-inflammatory cytokines, IL-8, IL-6 and TNF-α [12,40]. In addition, diminution of IL-6 levels was demonstrated when probiotic *Lactobacilli* strains were co-cultured with the pathogenic bacteria, *Escherichia coli* [4].

During an inflammatory reaction, the most important transcription factors of cytokine-mediated pro-inflammatory genes, e.g., IL-8, TNF-α, IL-1β, IL-6, are the nuclear factor κb (NF-κB) proteins family, which includes several genes (e.g., NF-κB1 and NF-κB2) and can be activated by LPS through specific receptors, called toll-like receptors 4 (TLR4). There are several pieces of evidence that the expression of TLR4 and then NF-κB genes is aberrant in chronic intestinal disease, which provokes strong inflammatory conditions, *i.e*., IBD. Contrarily, in healthy patients, the TLR4 and NF-κB expression is at very low levels [4]. As shown in Figure 4, the exposure of samples inoculated with *L. plantarum* strains to LPS-stimulated THP-1 cells indicated that the tested *Lactobacilli* inhibited the activation of the NF-κB1 gene, except for some treatments after 1 h of exposure (*in vitro* digested sample inoculated with *L. plantarum* CECT 8328). We speculate that a longer time of incubation could be necessary to determine a higher reduction of that gene expression. In fact, the increment of transcriptional level was also increased by LPS mainly after 4 h (around nine-fold) with respect to shorter incubation of 1 h (around two-fold).

Whereas the immune system contrasts the origin of injury or infection rousing the production of many pro-inflammatory molecules during an inflammatory process, the complex immune network, when it have removed the enemy, provides also the restoring of the immune homeostasis, inducinganti-inflammatory cytokines. The gene coding for interleukin 10 (IL-10) can mediate the downregulation of inflammatory progression [10]. In this study, we reported a relevant increase of the expression of IL-10 by probiotic bacteria for all *in vitro* digested samples after 1 h and for all treatments after 4 h of exposure. However, we noted a slight reduction of transcriptional level after 1 h of incubation by undigested samples, supposedly due to the presence of undigested yogurt matrix during the assay. Similar to our results, Cui *et al.* [41] studied the effects of probiotics on the intestinal mucosa of patients with ulcerative colitis (UC) and demonstrated that probiotics were able to enhance the expression of the anti-inflammatory cytokine IL-10 and to decrease the activation of NF-κB, reducing the expressions of TNF-α and IL-1β genes. The authors suggested the potential use of their probiotic preparation to contrast the flare-ups of chronic UC.

Lastly, we investigated the ability of the tested probiotics to influence the expression level of the thymic stromal lymphopoietin (TSLP) gene. TLSP is involved in the allergic response, *i.e*., bowel ulcerative colitis (UC) and Crohn’s disease, but also in asthma and dermatitis events. During those inflammatory processes, the transcriptional level of TSLP is upregulated [42,43]. TNF-α and IL-1β are able to control the induction of TSLP expression [44], but it may be alternatively increased via other pathways [43]. TSLP have been also shown to have a role in the tumor development of intestinal cells [45]. With respect to the TSLP gene, we found that the exposure of LPS-stimulated THP-1 cells to food matrix containing *L. plantarum* strains resulted in a decrease in the gene transcription occurring within 4 h, showing no increments of gene expression by treatments. Plausibly, the fact that probiotics may modulate the TSLP gene expression promotes a suitable application of these beneficial microbes as immune-modulators.

Comprehensively, the transcriptional analysis of cytokine-mediating genes showed that the probiotic bacteria used in this study have a favorable influence on immunomodulation. Overall, we did not find significant differences between undigested and *in vitro* digested treatments; thus, we concluded that the effects on immune stimulation were unrelated to digestive processes.

## 3. Experimental Section

### 3.1. Bacterial Strains, Human Cells and Growth Conditions

The bacterial strains used in this work were *Lactobacillus plantarum* CECT 8328, a strain already characterized for its probiotic features and ability to over-produce riboflavin in a co-culture system with Caco-2 cells [29], the probiotic *Lactobacillus plantarum* WCFS1 [46], *Streptococcus thermophilus* UFG24 and *Lactobacillus delbrueckii* ssp. *bulgaricus* UFG23, which were isolated from a homemade yogurt and identified by 16S ribosomal DNA amplification (data not shown).

*L. plantarum*, *S. thermophilus* and *L. delbrueckii* ssp. *bulgaricus* strains were propagated in de Man Rogosa Sharpe (MRS, Oxoid, Basingstoke, UK) (pH 6.2) and incubated at 30 °C (*L. plantarum* strains) and 42 °C (*L. delbrueckii* and *S. thermophilus* strains), respectively.

Human monocytoid leukemia-derived cells (THP-1) (Sigma-Aldrich, St. Louis, MO, USA) were grown in RPMI-1640 (Sigma-Aldrich) supplemented with 10% (v/v) FBS, 2 mM l-glutamine, 100 U/mL penicillin and 100 μg/mL streptomycin, in atmosphere containing 5% CO_2_ at 37 °C. Then, THP-1 cells were seeded at concentration of 5 × 10^5^ cells/well in 24-wells plates, resuspended in RPMI 1640 medium without any supplements and induced to differentiate into a mature macrophage-like state by treating for 48 h with 100 ng/mL phorbol 12-myristate 13-acetate (PMA) (Sigma-Aldrich).

### 3.2. Yogurt Fermentation

Yogurt was made from cow’s milk according to [47]. Briefly, the milk was heat-treated at 85 °C for 30 min, and the absence of microorganisms was ascertained by counting of CFU/mL on MRS agar plates. All bacteria strains, including *L. plantarum* WCFS1, *L. plantarum* CECT 8328, *S. thermophilus* and *L. delbrueckii* ssp. *bulgaricus*, were pre-incubated overnight, then centrifuged (2000× *g*, 10 min). The media was decanted, and the pellets were resuspended directly in milk to obtain a final concentration of 1 × 10^9^ CFU/mL. We carried out three different milk fermentations using the following combinations: (1) *S. thermophilus* and *L. delbrueckii* ssp. *bulgaricus* (positive control); (2) *S. thermophilus*, *L. delbrueckii* ssp. *bulgaricus* and *L. plantarum* WCFS1; and (3) *S. thermophilus*, *L. delbrueckii* ssp. *bulgaricus* and *L. plantarum* CECT 8328. All samples were then incubated at 42 °C for 6 h and then stored at 4 °C for 28 days. All trials were performed in duplicate.

### 3.3. Chemical Analysis

Milk samples were analyzed for fat, protein, lactose and casein content (MilkoScanTM, FT 120; Foss Electric, Hillerǿd, Denmark). Yogurt samples were analyzed after 1, 14 and 28 days of storage for protein, casein and nitrogen fractions by the Kjeldahl method according to the AOAC method [48] and for fat contents by the Gerber method according to the British Standards Institution [49]. The lactic acid content was detected by enzymatic kits according to the manufacturer’s instructions (Biogamma s.r.l, Roma, Italy).

For monitoring the hydrolysis of protein during storage of yogurt at 4 °C, the pH 4.6 water-soluble extracts (WSEs) of the samples were prepared according to the method proposed by Kuchroo and Fox [50].

The peptide profiles of the WSEs were determined by reverse-phase high performance liquid chromatography (RP-HPLC) using Agilent 1260 Infinity (Agilent Technologies, Santa Clara, CA, USA). The column used was a ZORBAX 300 SB-C18 (250 mm × 4.6 mm × 5 μm) (Agilent). The mobile phase was water (Solvent A) and acetonitrile (Solvent B), both containing 0.1% trifluoroacetic acid, and the solvent flow rate was 1 mL/min. The eluate was monitored at wavelength of 220 nm; all solvents were of chromatography grade (Baker, Inc., Phillipsburg, NJ, USA).

### 3.4. Microbiological Analysis

The viability of *L. plantarum* WCFS1 and *L. plantarum* CECT 8328 was monitored in all samples prior to fermentation (T0) and following fermentation after 1, 7 14, 21 and 28 days by quantitative real-time PCR (see below).

### 3.5. Oro-Gastrointestinal Tolerance Assay

Yogurt samples after 14 days of storage (as an intermediate shelf-life time) were exposed to a simulated oro-gastrointestinal transit, as described by Arena *et al.* [29]. Aliquots taken prior to theoro-gastrointestinal assay and after the oral, gastric (pH 2.0 and 3.0) and intestinal (small and large intestine sectors) stresses were used for the evaluation of bacterial survival, monitored by quantitative real-time PCR. The percentage of survival was determined with respect to the unstressed control. The samples, after the last step of intestinal tract stress (large intestinal compartment), were used for the stimulation of THP-1 cells assay.

### 3.6. Stimulation of THP-1 Cells with Lactobacilli

To perform the immunostimulation assay, both untreated (yogurt samples that were not exposed to the *in vitro* digestion assay) and *in vitro* digested yogurt samples were used. To decide the ratio of bacteria:human cells to be used, we considered two main aspects. Firstly, the percentage of cell survival after the entire *in vitro* digestion assay was around 1 × 10^6^ CFU/mL for *L. plantarum* WCFS1 and around 1 × 10^7^ CFU/mL *L. plantarum* CECT 8328. Secondly, the digestion solutions and enzymes used in the *in vitro* digestion can affect human cell viability; thus, the samples need to be diluted in a ratio of 1:3 (sample:medium) [51]. Hence, bacteria pellets were harvested by centrifugation (2000× *g* for 10 min) and resuspended in RPMI 1640 medium. The final concentration for *L. plantarum* WCFS1 was 3 × 10^5^ CFU/mL (both for untreated and *in vitro* digested samples), and for *L. plantarum* CECT 8328, this was around 3 × 10^6^ CFU/mL (both for untreated and *in vitro* digested samples).

Macrophage-differentiated THP-1 cells were treated as described elsewhere [19]. Briefly, THP-1 cells were exposed to 100 ng/mL of lipopolysaccharide (LPS) from *E. coli* O127:B8 (Sigma). Our samples (untreated and *in vitro* digested samples appropriately diluted) were added and incubated for 1 and 4 h at 37 °C with 5% CO_2_. Positive and negative controls were macrophage-differentiated THP-1 cells incubated with and without LPS, respectively. Human cells were harvested, and transcriptional analysis was performed for genes coding immune-related genes (see below).

### 3.7. Propidium Monoazide Treatment and Microbial DNA Extraction

Yogurt samples were treated with propidium monoazide (PMA), as previously described by Àlvarez *et al.* [27]. Briefly, 100 μM of PMA (Biotium, Inc., Hayward, CA, USA) dissolved in 20% of dimethylsulfoxide (DMSO) (Sigma) were added to 1 mL of yogurt samples and kept in light-transparent 1.5-mL microcentrifuge-tubes. The tubes were incubated in dark conditions for 10 min and then exposed to a halogen lamp (650 W, 230 V, GY 9.5, 3050 K; Philips, Tokyo, Japan).

Successively, the genomic DNA of each strain was extracted from the yogurt samples pre-treated by PMA, as reported by Quigley [52], using a lytic method. Briefly, 1 mL of each sample was added to 0.5 mL of breaking buffer for enzymatic lysis and incubated at 37 °C for 1 h. Subsequently, the samples were treated with proteolytic enzyme by adding 250 μg/mL of proteinase K and incubating at 55 °C for 1 h. The suspension was transferred to a new tube containing zirconium beads, shaken twice for 90 s and centrifuged at 12,000× *g* for 10 min. The supernatant was added to an equal volume of phenol:chloroform:isoamyl alcohol (25:24:1), mixed gently and centrifuged at 12,000× *g* for 2 min. Only the upper aqueous phase was transferred into a clean tube. Sodium acetate (3 M) (one-tenth the volume) and 100% ice-cold ethanol (2 volumes) were added. The samples were mixed, stored at −20 °C overnight and then centrifuged at 14,000× *g* for 10 min in order to harvest the pellet that was washed with 70% ice-cold ethanol followed by centrifugation at 12,000× *g* for 5 min and dried. The final pellet was resuspended in 100 μL TE buffer and used in Q-PCR detection.

### 3.8. THP-1 RNA Extraction and Reverse Transcription

RNA extraction, cDNA synthesis and quantitative RT-PCR were performed as previously described [53]. Briefly, THP-1 macrophages were harvested with TRIzol reagent (Invitrogen, Carlsbad, CA, USA). The total RNA was extracted according to the manufacturer’s instructions. The RNA concentration and integrity were determined by spectrophotometry (Biotek Instruments, Winooski, VT, USA) and gel electrophoresis. One microgram of total RNA was reverse-transcribed using the QuantiTect Reverse Transcription kit (Qiagen, Valencia, CA, USA).

### 3.9. Q-PCR Analysis

The extracted microbial DNA were diluted (1:20), and 5 μL were used to perform the Q-PCR analysis (ABI 7300, Applied Biosystems, Foster City, CA, USA) in a reaction mixture containing 15 μL of PCR mix (Power SYBR Green PCR Master Mix; Applied Biosystems) and 100 nM of forward and reverse primers for gyrA amplification specific for *L. plantarum* species [54]. Serial dilutions of known *L. plantarum* WCFS1 DNA, in amounts ranging from 1 × 10^4^ to 1 × 10^8^ CFU/mL, were carried out to generate a reference standard curve, which was used for the relative quantification.

The obtained human cDNA samples were also diluted (1:20), and 5 μL were used to perform the Q-PCR analysis. Primers (100 nM of each primer) were selected from PrimerBank [55]. Primers related to glyceraldehyde phosphate dehydrogenase (GAPDH), β-actin (β-actin), interleukin 8 (IL-8) and interleukin 6 (IL-6) genes were previously reported by Bove *et al.* [53]. Primers for the gene tumor necrosis factor α (TNF-α) were designed (Table 2). GAPDH, β-actin and hypoxanthine phosphoribosyl transferase 1 (HPRT1) genes were used to normalize the expression of target genes by the 2^−ΔΔCt^ method [56].

The thermal conditions were 95 °C for 10 min followed by 40 cycles of 95 °C for 20 s, 58 °C for 30 s and 72 °C for 30 s. Each PCR assay included duplicate reactions.

**Table 2:** Oligonucleotides used in Q-PCR analysis.

Oligonucleotide	Name	Sequence (5′–3′)
IL-1βF	Interleukin 1β	ATGATGGCTTATTACAGTGGCAA
IL-1βR		GTCGGAGATTCGTAGCTGGA
NF-κB1F	Nuclear factor kappa B	GGTGCGGCTCATGTTTACAG
NF-κB1R		GATGGCGTCTGATACCACGG
IL-10F	Interleukin 10	GACTTTAAGGGTTACCTGGGTTG
IL-10R		TCACATGCGCCTTGATGTCTG
TSLPF	Thymic stromal lymphopoietin	ATGTTCGCCATGAAAACTAAGGC
TSLPR		GCGACGCCACAATCCTTGTA
GAPDHF	Glyceraldehyde phosphate dehydrogenase	CGACCACTTTGTCAAGCTCA
GAPDHR		AGGGGTCTACATGGCAACTG
β-actF	β-Actin	AAAGACCTGTACGCCAACAC
β-actR		CATACTCCTGCTTGCTGATCC
IL-6F	Interleukin 6	TACCCCCAGGAGAAGATTCC
IL-6R		TTTTCTGCCAGTGCCTCTTT
IL-8F	Interleukin 8	TGTGGAGAAGTTTTTGAAGAGGG
IL-8R		CCAGGAATCTTGTATTGCATCTGG
TNF-αF	Tumor necrosis factor α	AACCTCCTCTCTGCCATCAA
TNF-αR		ATGTTCGTCCTCCTCACAGG

### 3.10. Statistical Analysis

Each datum represents the mean ± SD of two biological experiments and three technical replicates. Data were analyzed via principal components analysis (PCA) and the Student’s *t*-test using the PAST version 2.17C software program [57]. *p* < 0.05 and *p* < 0.005 were considered as statistically significant.

## 4. Conclusions

We used two *L. plantarum* strains to produce yogurt in order to analyze their technological and probiotic features, focusing on the potential immunomodulatory ability. The yogurts obtained were shown to have: (i) a chemical profile similar to that of the control yogurt in terms of lactic acid, nitrogen fractions and fat content; (ii) a different profile of peptide content; and (iii) good stability over 28 days of storage. Moreover, the viability of inoculated strains was persistent over the shelf-life, and all *L. plantarum* strains inoculated into the yogurt matrix were able to tolerate the oral, gastric and intestinal stress conditions. The strain, *L. plantarum* CECT 8328, performed better compared to *L. plantarum* WCFS1. Finally, the transcriptional analysis of genes involved in the immune response showed that the studied probiotic bacteria possibly have a positive influence on immunomodulation, and this feature is not affected during the human digestion process. In summary, the tested *Lactobacilli* exhibited suitable technological features for yogurt production, and we suggest that they might be used as immunomodulating strains to formulate novel foods with beneficial effects on immune homeostasis.
